# Sugars speed up the circle of life

**DOI:** 10.7554/eLife.00625

**Published:** 2013-03-26

**Authors:** Marcel Proveniers

**Affiliations:** 1 **Marcel Proveniers** is at the Molecular Plant Physiology group, The Institute of Environmental Biology, Utrecht University, Utrecht, The Netherlandsm.proveniers@uu.nl

**Keywords:** microRNA, developmental timing, phase change, sugar, heteroblasty, plant biology, Arabidopsis

## Abstract

By regulating the expression of key microRNA molecules, sugar levels in leaves control the transition from the juvenile to the adult form in plants.

**Related research articles** Yang L, Xu M, Koo Y, He J, Poethig RS. 2013. Sugar promotes vegetative phase change in *Arabidopsis thaliana* by repressing the expression of *MIR156A* and *MIR156C*. *eLife* **2**:e00260. doi: 10.7554/eLife.00260; Yu S, Cao L, Zhou C-M, Zhang T-Q, Lian H, Sun Y, Wu J, Huang J, Wang G, Wang J-W. 2013. Sugar is an endogenous cue for juvenile-to-adult phase transition in plants. *eLife* **2**:e00269. doi: 10.7554/eLife.00269

**Image** Gene expression studies in *Arabidopsis* reveal how sugars regulate a key developmental transition in plants

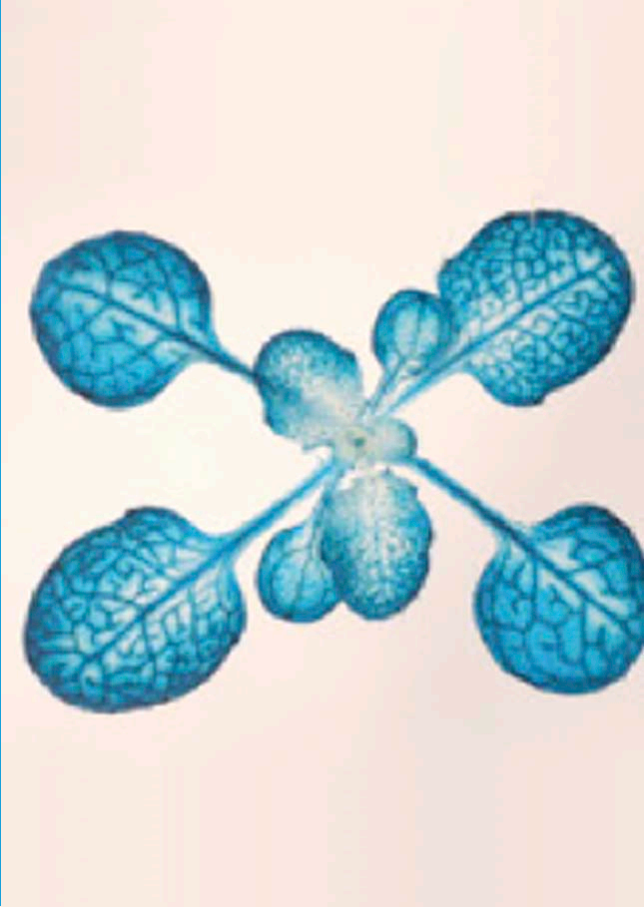

How do plants determine which stage of development they are at? This is a long-standing and important question in plant biology. Plants, like other higher organisms, progress through a series of developmental phases, and the correct timing of these phase changes is critical for growth and reproduction, and thus for survival in the natural environment.

After a seed has germinated, the shoot progresses through a juvenile vegetative phase, an adult vegetative phase and ultimately, when conditions are right, a reproductive phase (Figure 1). The juvenile vegetative phase has a critical role in plant development. Juvenile plants rapidly increase their photosynthetic capacity, size and mass to ensure maximum productivity, but are incapable of initiating reproductive development. The transition from the juvenile vegetative to the adult vegetative stage, during which plants acquire reproductive competence, is known as vegetative phase change. During this transition, changes in multiple traits, including leaf size and shape, and leaf trichome (hair) distribution, result in the appearance of both juvenile and adult traits on the same plant, a condition known as heteroblasty.

**Figure 1.**
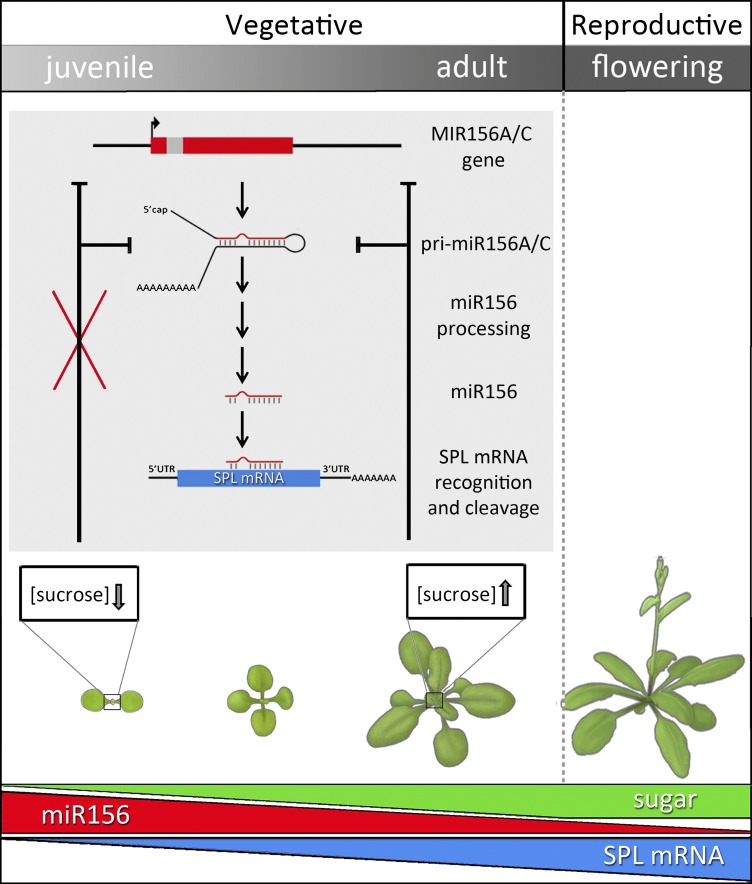
After germination, plants enter a juvenile vegetative phase and then transition to an adult vegetative phase before producing reproductive structures. In *Arabidopsis*, the onset of the adult phase is characterized by the appearance of hairs on the lower surface of the leaf and a change in leaf shape from round leaves with smooth edges to elongated leaves with serrated edges. The juvenile-to-adult transition, or vegetative phase change, is controlled by the activity of microRNA-156. The predominant genes encoding this miRNA—*MIR156A* and *MIR156C*—are transcribed to produce primary mRNA transcripts (pri-miR156 A/C), which are further processed to generate mature transcripts (miR156). These guide the cleavage of target (SPL) mRNAs, thereby reducing SPL mRNA abundance, which has the effect of preventing vegetative phase change. Sugars regulate the timing of the juvenile-to-adult transition by repressing miR156 accumulation. Early in development, miR156 levels are high, promoting juvenile traits. As the plant matures, miR156 levels steadily decrease (red bar). After germination, plants start to accumulate sugars through photosynthesis and, as they grow older, the overall photosynthetic output of the shoot increases (green bar). Sucrose, the major transportable sugar, moves from the pre-existing leaves to the young leaf primordia (boxed), where its accumulation inhibits the transcription of *MIR156A* and *MIR156C*. In the initial steps of glycolysis, sucrose is broken down into glucose, which in turn causes degradation of the miR156 precursors pri-miR156A and C. As a result, miR156 levels decrease and SPL levels increase (blue bar), thereby promoting the expression of adult traits.

Previous studies have shown that, as a plant ages, a gradual decline in abundance of the evolutionarily conserved microRNA-156 (miR156) promotes the expression of adult traits (reviewed in Huijser and Schmid, 2011). However, the nature of the age-dependent factor(s) that promote the decline in miR156 levels remained a mystery. Now, writing in *eLife*, Li Yang, Scott Poethig and co-workers at the University of Pennsylvania (UPenn; Yang et al., 2013) and, independently, Sha Yu and Jia-Wei Wang of the Shanghai Institute of Plant Physiology and Ecology and their colleagues take a key step towards answering this question (Yu et al., 2013). Both groups identified sugar produced by photosynthesis as an endogenous developmental timing cue for the juvenile-to-adult phase transition, with the sugar acting to modulate miR156 expression.

Previous research by the UPenn group indicated that vegetative phase change is mediated by a factor (or factors) produced in leaves and that this factor (or factors) acts by repressing the expression of miR156 in young leaf primordia (Yang et al., 2011). For decades there have been indications that sugars, as products of photosynthesis, are important for shoot maturation. Given the critical role of sugars as signaling molecules in plants, in addition to their functions in metabolism (reviewed in Smeekens et al., 2010), both groups investigated the role of sugar in vegetative phase change. They used a mutant form of the model plant *Arabidopsis* with low efficiency photosynthesis (the *chlorophyll a oxygenase*/*chlorina1* plant) to demonstrate that a reduced rate of photosynthesis delays the juvenile-to-adult phase transition. Moreover, it does so in an miR156-dependent manner since mutant plants that additionally lack miR156 activity do not show the delayed phase change phenotype.

The two groups then studied the effect on miR156 expression and vegetative phase change of supplying the plants with exogenous sugar, and found that glucose, fructose, sucrose and maltose repressed the accumulation of miR156. This is not the result of osmotic stress (that is, a disturbance in the water balance of the plant due to application of a sugar solution that is more concentrated than the intracellular solution), as metabolically inactive (but osmotically equally active) sugars did not repress miR156 levels. miR156 inhibits the expression of genes encoding SPL proteins—a family of transcription factors that promote developmental transitions—by targeting SPL mRNAs for cleavage (Figure 1; Wu and Poethig, 2006; Wang et al., 2009). Accordingly, transcript levels of *SPL9* and *SPL15* (Yu et al., 2013) increased markedly upon addition of sugar, as did those of *SPL9-GUS*—a gene fusion of *SPL9* and the reporter gene *GUS*. However, sugar had no effect on the expression of an *SPL9-GUS* fusion transcript that lacked a functional miR156-responsive element (Yang et al., 2013). The finding that sucrose application increased the proportion of leaves with hairs on their lower surface (Yang et al., 2013)—a morphological mark for the onset of the adult phase—further supports the conclusion that the effect of sugar on miR156 and *SPL* expression has functional significance.

In *Arabidopsis*, there are eight genes encoding miR156 (*MIR156A-H*) (Reinhart et al., 2002). The UPenn and Shanghai groups show that *MIR156A* and *MIR156C* are the predominant regulators of vegetative phase change, and that sugar treatment strongly downregulates the expression of both genes in young leaf primordia. Intriguingly, glucose and sucrose each seem to regulate *MIR156A* and *MIR156C* expression by different mechanisms. Both these genes are first transcribed to produce primary mRNA transcripts (pri-miR156 A/C), which in turn are processed to generate mature transcripts (miR156). The UPenn group reports that glucose, in contrast to sucrose, has little or no effect on the expression of *MIR156A-GUS* and *MIR156C-GUS—*fusion transcripts in which *MIR156A* and *MIR156C* hairpin sequences have been replaced with the reporter gene *GUS*—whereas both sugars significantly reduced the abundance of the endogenous *pri-MIR156A* and *pri-MIR156C* transcripts in these reporter lines. These results suggest that sucrose regulates the transcription of *MIR156A* and *MIR156C* to produce primary transcripts, whereas glucose regulates the expression of these genes by a post-transcriptional mechanism. In line with this, the Shanghai group reports that glucose represses the accumulation of *pri-MIR156A* and *pri-MIR156C* transcripts even in the presence of a transcriptional inhibitor, suggesting that glucose modulates miR156 expression levels through degradation of these primary miRNAs (Yu et al., 2013).

Both studies clearly demonstrate that sugars regulate shoot maturation in *Arabidopsis* by repressing the expression of miR156 in leaves. However, a key question that remains is whether sugars are the leaf-derived signal that controls vegetative phase change. Defoliation experiments in *Arabidopsis* (Yu et al., 2013) and tobacco plants (Yang et al., 2011, 2013) show that removing the two oldest leaves results in increased miR156 levels in shoot apices and a delay in the expression of adult-specific traits. The fact that sugar application partially reverses this effect indicates that sugars derived from photosynthetically active leaves are likely candidates for the long-distance signals that repress miR156 expression in young leaf primordia. However, it remains to be determined whether endogenous sugar levels do increase in these young primordia during vegetative phase change. In addition, one can ask whether endogenous sugars fulfill the same role in species that undergo a much longer juvenile phase than *Arabidopsis*, such as trees and other perennial woody plants, where vegetative phase change occurs after months or even years.

The work of the UPenn and Shanghai groups indicates that sucrose and glucose affect miR156 levels via different mechanisms. Elucidating the rationale behind such a complex mode of regulation and dissecting the underlying molecular mechanism(s) will be major challenges. Recent work by Vanessa Wahl and co-workers at Max Planck Institutes in Potsdam and Tubingen revealed that, in shoot meristems (organized clusters of cells that generate the above-ground parts of the plant), a molecule called trehalose-6-phosphate (T6P) acts as a local signal that links sugar availability to developmental decisions (Wahl et al., 2013). Moreover, T6P controls the expression of *SPL* genes partially via the age-dependent miR156 pathway. It will be worthwhile investigating whether this sugar-miRNA156 pathway is also involved in the juvenile-to-adult phase transition in young leaf primordia. Despite these questions, the results presented by the UPenn and Shanghai groups provide the first molecular evidence for the long-standing hypothesis that shoot maturation is regulated by an increase in endogenous carbohydrates.
